# Amyloidogenesis and Neurotrophic Dysfunction in Alzheimer’s Disease: Do They have a Common Regulating Pathway?

**DOI:** 10.3390/cells11203201

**Published:** 2022-10-12

**Authors:** Fengjuan Jiao, Dongjun Jiang, Yingshuai Li, Juan Mei, Qinqin Wang, Xuezhi Li

**Affiliations:** 1 Shandong Collaborative Innovation Center for Diagnosis, Treatment and Behavioral Interventions of Mental Disorders, Institute of Mental Health, Jining Medical University, Jianshe South Road No.45, Rencheng District, Jining 272013, China; 2 Shandong Key Laboratory of Behavioral Medicine, School of Mental Health, Jining Medical University, Jianshe South Road No.45, Rencheng District, Jining 272013, China

**Keywords:** Alzheimer’s disease, amyloid-β, amyloidogenesis, neurotrophic

## Abstract

The amyloid cascade hypothesis has predominately been used to describe the pathogenesis of Alzheimer’s disease (AD) for decades, as Aβ oligomers are thought to be the prime cause of AD. Meanwhile, the neurotrophic factor hypothesis has also been proposed for decades. Accumulating evidence states that the amyloidogenic process and neurotrophic dysfunction are mutually influenced and may coincidently cause the onset and progress of AD. Meanwhile, there are intracellular regulators participating both in the amyloidogenic process and neurotrophic pathways, which might be the common original causes of amyloidogenesis and neurotrophic dysfunction. In this review, the current understanding regarding the role of neurotrophic dysfunction and the amyloidogenic process in AD pathology is briefly summarized. The mutual influence of these two pathogenesis pathways and their potential common causal pathway are further discussed. Therapeutic strategies targeting the common pathways to simultaneously prevent amyloidogenesis and neurotrophic dysfunction might be anticipated for the disease-modifying treatment of AD.

## 1. Introduction

Alzheimer’s disease (AD) is among the most common neurodegenerative disorders of the central nervous system in the elderly. The major neuropathological hallmarks of AD are amyloid plaques comprised of amyloid-β (Aβ) peptides, neurofibrillary tangles (NFTs) primarily composed of hyperphosphorylated tau proteins, selective basal forebrain cholinergic neuron (BFCN) degeneration and brain atrophy. Among the hypotheses for AD pathogenesis, the amyloid cascade hypothesis has dominated in the last few decades [1], and postulates that the abnormal accumulation of amyloid plaques causes neurodegeneration in various areas of the brain. Thus, Aβ oligomers are proposed to be the prime cause of AD, and toxic Aβ oligomer accumulation trigger the other pathologies, such as tau pathology, and inflammatory and synapse damage.

Another classic explanation for the pathogenesis of AD is the neurotrophic factor hypothesis [2,3], represented by the nerve growth factor (NGF) hypothesis [4,5]. Both laboratory and clinical research suggest that the neurotrophic status, supported by NGF and other neurotrophic factors (NTFs), plays a critical role in AD progression [6,7]. In the adult brain, enriched NGF expression appears to be restricted to few areas, of which the basal forebrain cholinergic system (BFCS) is representative. The BFCN plays a critical role in cognitive behavior and attention behavior. In addition to the classic characteristic changes observed in other neuronal systems and the presence of neuronal plaques and tangles, cholinergic deficits in the BFCN seem to be a principal element responsible for the memory loss typical of AD [8]. Significant cholinergic dysfunction and cholinergic neuronal degeneration are associated with cognitive deficits in AD [9,10].

The exact causes of AD are not fully understood and there is still no disease-modifying treatment that cures the disease or alters the disease process. Most of the theories, including the amyloid cascade hypothesis, tau hypothesis and inflammation hypothesis, explain AD pathogenesis from the perspective of overloaded toxicity or stress, whereas amyloidogenesis pathology seems more specific to AD and is better characterized. Complementary to this, the neurotrophic factor hypothesis tries to clarify AD pathogenesis in consideration of the shortage of neurotrophic outcomes. New theories, such as the traffic jam hypothesis, also suggest that upregulated amyloidogenesis and downregulated neurotrophic factor signaling mutually drive AD pathogenesis [11].

As both amyloidogenesis and NTFs are in charge of AD pathogenesis, balancing their neurotoxic and neurotrophic effects may be a considerable strategy for preventing AD. The neurotoxic roles of Aβ oligomers and the neurotrophic roles of NTFs on AD pathogenesis have been extensively reviewed [12,13,14,15,16]. In fact, the signaling pathway of NTFs can regulate amyloidogenesis [17,18], and proteins in the amyloidogenic process pathway affect the NTF signaling pathways [19,20]. Here, the current understanding of the role of NTFs and amyloidogenesis, as well as their interactions, in AD pathology are briefly summarized. The factors linked with both neurotrophic dysfunction and the amyloidogenic process in the pathology of AD are discussed. It is possible that there are various instances of co-operation between each well-characterized pathogenesis of AD that are interesting. It would be meaningful for these to be illustrated in the future to better understand AD pathology and treatment.

## 2. Amyloidogenesis and AD

The best-characterized histopathology hallmark in the AD brain is the extracellular plaques comprised of the Aβ peptides, which are proteolytic fragments of the amyloid precursor protein (APP) cleavage by β-secretases and γ-secretases. Generally, APP can be processed via α secretase (A disintegrin and metalloproteinases 10/17 (ADAM 10/17)) as a physiological pathway to begin the generation of α products (soluble APPα (sAPPα), c-terminal fragment CTFα), or via β secretase (β-site amyloid precursor protein cleaving enzyme-1 (BACE1) is the major one) as amyloidogenic pathways to begin the generation of β products (soluble APP β (sAPPβ) and C-terminal fragment CTFβ/C99) [21,22]. The cleavage of APP by BACE1 is the rate-limiting step in the generation of Aβ peptides, creating the C99 fragment that becomes a substrate for subsequent γ-secretase (consisting of presenilin, nicastrin, APH-1 and PEN2) to generate mature Aβ peptides. APP cleavage by BACE1 and γ-secretase both occur at the cell membrane and endocytic compartments, respectively. Combining APP cleavage by BACE1 with its processing within compartments of the endocytic pathway results in the overproduction of Aβ products and is proposed to be a pathological mechanism for AD [23].

The earliest Aβ aggregate appears in the parietal lobe, medial temporal lobe and frontal lobe, and then, it gradually exists in the entire neocortex, diencephalon, corpus striatum and more areas [24]. Accumulated clinical and laboratory research supports the assumption that amyloid plaques and soluble amyloid aggregates are the upstream cause of AD, and subsequently trigger the other pathologies, forming the amyloid cascade hypothesis. The general concept that Aβ is associated with cognitive impairment is confirmed by an 11-year follow-up study [25] and a meta-analysis study [26]. Progress in the Aβ-related pathobiology of AD has been extensively documented recently [14]. The amyloid cascade hypothesis is also facing challenges. The development of nearly all drugs targeting amyloid pathology has failed.

## 3. NTFs and AD

NTFs are a kind of growth factor secreted by neurons, glial cells and target tissues that promote the growth, differentiation and survival of nerve cells during the development and maintenance of the nervous system [27]. The hypothesis of NTFs in AD supports the idea that AD is caused by a deficiency in neurotrophic pathways, making NTF targeting a potential therapeutic tool for AD. The anticipated roles of NTFs in AD treatment have been discussed recently by various labs [28,29,30,31]. Most NTFs exert their effects via signaling through specific transmembrane receptor tyrosine kinases (RTKs) and are typically classified into the following groups: (1) Neurotrophins, the most-studied family of NTFs. The neurotrophins include NGF, brain-derived neurotrophic factor (BDNF) and neurotrophin-3/4/5 (NT-3/4/5). They can be associated with the corresponding high-affinity tropomyosin-related kinase receptors (Trks) to induce their tyrosine kinase activity and downstream signal transduction. NGF specifically binds to the TrkA receptor, BDNF and NT-4/5 specifically binds to the TrkB receptor, and NT-3 specifically binds to the TrkC receptor. Additionally, they can bind to the low-affinity p75 neurotrophic factor receptor (p75NTR) and induce apoptosis. (2) Glial cell-line-derived neurotrophic factor family ligands (GFLs), consisting of glial cell-line-derived neurotrophic factor (GDNF), neurturin (NRTN), artemin (ARTN) and persephin (PSPN). GFLs signal via a multicomponent receptor system consisting of a high-affinity ligand-binding glycosyl-phosphatidylinositol (GPI)-linked co-receptor (GFRα1-4) and the receptor tyrosine kinase (RET). GDNF, NRTN, ARTN and PSPN interact mainly with GFRα1, GFRα2, GFRα3 and GFRα4, respectively, to recruit and activate RET. (3) Neuropoietic cytokines, which are a big group of small secreted proteins, signal through a gp130 receptor complex, consisting of ciliary neurotrophic factor (CNTF), leukemia inhibitory factor (LIF), etc. (4) Evolutionarily conserved cerebral dopamine neurotrophic factor (CDNF) and mesencephalic astrocyte-derived neurotrophic factor family (MANF). Besides the “classical” neurotrophic factors, there are growth factors with neurotrophic effects and newly classified NTFs, such as vascular endothelial growth factor (VEGF) and neurotrophic factor-α1 (NF-α1, also known as carboxypeptidase E or CPE), that have also been linked to AD [32,33]. The NTFs and growth factors which have altered expressions or mutations in AD patients are shown in Table 1.

NGF was the first identified NTF and is one of the most researched NTFs associated with AD. Original research exploring the roles of NTFs on AD was initiated in NGF and has accordingly transitioned to clinical trials [68,69]. Dysfunction of the NGF pathway is tightly linked with profound and early BFCN degeneration [8,70,71,72,73]. Studies have proved that the expression of TrkA, but not p75NTR, is downregulated in the BFCNs and the cortex of AD patients [74,75], while the NGF-immunoreactive protein (which is now thought to be proNGF, the NGF precursor protein) level is elevated in the cortex and hippocampus and degraded in the basal forebrain [37,76,77]. This indicates that the retrograde transport of NGF mediated by TrkA is deficient, and hence, inadequate of neurotrophic signaling to maintain neuron survival [78,79]. It is now verified that proNGF is the major species in the adult human brain, whereas mature NGF is completely absent [80,81,82]. Indeed, proNGF is more sensitive to the balance of TrkA and p75NTR levels. proNGF acts as an NTF by activating TrkA-dependent signaling pathways in BFCNs that express normal levels of TrkA and p75NTR [70,80,83]. The decrease in TrkA levels might disturb the balance between TrkA and p75NTR for proNGF binding and result in increased proNGF/p75NTR interaction to induce apoptosis. Thus, the lack of survival signaling and the exacerbation of apoptosis signaling eventually promote neurodegeneration. BDNF is a key molecule to maintain hippocampal synaptic plasticity and memory storage which are damaged in AD. The deficits of BDNF have been linked with Aβ accumulation, tau phosphorylation, neuroinflammation and neuronal apoptosis, and the roles of the BDNF pathway in AD have been well-reviewed recently [84,85]. GDNF is critical for the development, survival and maintenance of midbrain dopaminergic neurons. The exogenous expression of GDNF in the astrocytes of aged rats was able to improve their cognitive deficits, and GDNF administration had a protective role against AD-like changes in animal models [86]. A recent study suggested that NT-3 promotes the neuronal differentiation of bone marrow-derived mesenchymal stem cells (proposed to be an effective therapy for neurodegenerative diseases, including AD) and improved cognitive function in an AD rat model [87].

## 4. Mutual Influence of Amyloidogenic Process and Neurotrophic Pathways

There is an interaction between the amyloidogenic process and neurotrophic pathways. Increased Aβ levels in AD trigger tau pathology; this leads to microtubule deficits and related disorders, such as defective microtubule assembly and axonal transport deficits, which are likely to affect the neurotrophic signaling of NTFs, followed by neuronal death, and eventually, disease [88,89]. Aβ can bind to p75NTR, resulting in impaired p75NTR polyubiquitination, TRAF6/p62/p75NTR interaction and NF-κB activation and inducing neuronal cell death [90,91]. Furthermore, Aβ abrogates the NGF-induced tyrosine phosphorylation and polyubiquitination of TrkA in PC12 cells and human hippocampal tissues, and hence, deactivates the downstream Ras/MAPK and PI3K/Akt signaling pathways [92]. Aβ decreases BDNF expression through variable pathways [93,94], and Aβ inhibits the expression level of the full-length TrkB receptor and the ability of BDNF to modulate neurotransmitter (GABA and glutamate) release and long-term potentiation in vitro [95,96]. On the other hand, the NGF and BDNF signaling pathways modulate the amyloidogenic route and Aβ production in the cultured hippocampal neurons of rats [17,97]. NGF deprivation in animal models results in Aβ accumulation/deposition, while NGF treatment ameliorates Aβ pathologic changes [98,99,100,101]. NGF administration upregulates the expression of ADAM10 and two enzymes (disintegrin and metalloprotease-17) with α-secretase activity and downregulates BACE1 expression, driving APP cleavage towards the non-amyloidogenic pathway [102,103,104]. NGF also regulates microglial homeostatic activities and prevents Aβ accumulation pathologies via its anti-inflammatory activity in the microglia [105].

Both in vitro and in vivo studies indicate that APP and the NGF/TrkA signaling pathway are interconnected. Furthermore, the APP protein exhibits direct binding with TrkA. The APP phosphorylation pattern is considered as a potential therapeutic target for AD as it determines APP binding to cytosolic interactors and intracellular trafficking, which will finally affect APP processing [20,106,107,108,109]. NGF stimulation promotes TrkA/APP interaction by increasing APP phosphorylation at Y682 and reducing APP phosphorylation at T668, to facilitate the transport of APP to the Golgi apparatus [110], to disturb APP interactions with BACE1 [111], to regulate TrkA activation and subcellular distribution [20,112]. Finally, it inhibits APP processing to Aβ and enhances NGF neurotrophic action. The regulation of APP phosphorylation by NGF and subsequent processes are potentially disrupted in AD patient brains, where the phosphorylation level of APP T668 is increased and the APP/TrkA interaction is reduced [111]. In fact, APP/TrkA interactions are present in brain tissues from normal rat, mouse, and human, but not in brain tissues from AD groups [20,111,112]. The aging pathway decreases TrkA expression levels, results in a TrkA-to-p75NTR receptor switch for NGF signaling and leads to Aβ peptide generation, potentially explaining why aging is a risk factor for AD [113,114,115]. NGF signaling through p75NTR increases both ceramide levels (it can regulate both the α- and β-cleavage of APP) and the steady-state levels of BACE1 and CTFβ, which is NGF dose-dependent and specific for p75NTR, not for TrkA [115]. Another study found that p75NTR upregulated BACE1 transcription and enhanced BACE1 activity on APP by activating c-Jun N-terminal kinase (JNK) in SHSY5Y cells [116].

## 5. Is There Common Pathway to Control Both the Amyloidogenic Process and the Neurotrophic Pathways?

Although it is clear that amyloidogenesis and neurotrophic dysfunctions are mutually influenced, it is hard to figure out which comes earlier, and whether they work independently, coordinately, simultaneously or successively in AD development. Soluble toxic Aβ oligomers, not the amyloid fibrils, are thought to be the principal pathogenic Aβ species in AD and can be identified in human cerebrospinal fluid (CSF) decades prior to AD onset [1,117]. BDNF mRNA levels and NGF metabolism are also dysregulated early in the pre-clinical stage of AD [7,118]. In addition to the most-studied rare risk genes directly participating in amyloidogenesis (such as *APP*, *PSEN1* and *PSEN2*) for AD, genetic studies have identified dozens of risk genes, which can mostly be classified as participants in the endosomal trafficking pathways, the innate immune response pathways and the cholesterol metabolism pathways [119].

Both genetics and pathology started suggesting endosomal abnormalities and dysfunction as an early etiology in AD pathogenesis decades ago [120,121]. Furthermore, the recently developed traffic jam hypothesis proposes that the endosomal trafficking pathway is the universal cause in the multiple pathologies of AD [11,119,122]. According to the hypothesis, age-dependent endocytic trafficking dysfunction can alter Aβ production and clearance, neurotrophic signaling, etc. These alterations coordinately function in AD progression. In fact, among the products of these identified risk genes, there are regulators participating both in the control of amyloidogenic process and in the neurotrophic pathways, suggesting that amyloidogenesis and neurotrophic dysfunctions share common regulators, and thus, the common causal pathogenesis of AD. The regulators involved in controlling both amyloidogenesis and neurotrophic signaling are listed in Table 2. ApoE4, SORLA, sortilin, GGA3 and BIN1 can directly interact with targets (BACE1, APP, Aβ or TrkA et al.), whereas Arf6, CD2AP, retromers (including VPS35, VPS26 and VPS29) and Rab proteins act as the subsequent effectors in intracellular vesicle transport.

Apolipoprotein E (ApoE) is a kind of 34-kDa glycoprotein acting as cholesterol transporter to mediate the binding of lipoproteins or lipid complexes to specific cell-surface receptors. Of the three major ApoE isoforms (ApoE2, apoE3 and apoE4), ApoE4 has been identified as a major risk factor for AD, and the underlying mechanisms are widely investigated. ApoE4 controls Aβ aggregation, clearance, degradation, etc. via interactions with Aβ at multiple stages [123], while it suppresses BDNF mRNA expression by upregulating the nuclear translocation of histone deacetylases (HDACs) [93]. Thus, the high levels of ApoE4 in many AD patients may strengthen both Aβ neurotoxicity and the lack of BNDF neurotrophic signaling.

The sorting-related receptor with type-A repeats (SorLA, also named SorL1 or LR11) and sortilin are both vacuolar protein-sorting 10 protein (VPS10p) domain receptors involved in pleiotropic functions in intracellular cargo trafficking and signaling. SorLA has been linked tightly with APP cleavage by promoting APP trafficking away from the endosome, attenuating APP oligomerization, or inhibiting APP/BACE1 interactions [128,129,130,131,132,133]. SorLA directs the lysosomal targeting of Aβ peptides by binding with Aβ. SorLA also controls the sorting of GDNF and its receptors (GFRα1/RET) to regulate the subsequent neurotrophic activity [135], mediates the trafficking of TrkB to enhance the response of neurons to BDNF [136], and controls the activation of the EGFR/ERK/Fos signaling pathway to regulate neurite outgrowth and regeneration [137]. Sortilin facilitates BACE1 retrograde trafficking to the Golgi body to increase the cleavage of APP to Aβ [140,141], targets APP for lysosomal degradation and promotes APP cleavage by α-secretase for non-amyloidogenic processes [142]. Conversely, sortilin binds extracellular ApoE/Aβ complexes to facilitate their delivery to lysosomes for degradation [143,144]. Sortilin participates in the toxic effects of Aβ oligomers, as it acts as a receptor for oligomerized Aβ to mediate its endocytosis and induce apoptosis [145]. Sortilin also acts as receptor for ligands such as mature neurotrophins and proneurotrophins, known as neurotensin receptor-3 (NTR3) [149]. Sortilin interacts with Trk receptors to facilitate anterograde transport and neurotrophic signaling [146]. It can also serve as a co-receptor governing p75NTR binding with proNGF to induce cell death and neurodegeneration, acutely and chronically [147,148].

The Golgi-associated, gamma adaptin ear-containing, Arf-binding (GGA) proteins belong to a family of proteins that function as clathrin adaptors during intracellular vesicle trafficking. GGA1 and GGA3 control BACE1 degradation, recycling and axon transport, thus regulating BACE1 location and activity [151,152,153]. GGA3 also regulates the NGF pathway by enhancing TrkA post-endocytic recycling [154] or by rapidly recruiting p75NTR to the plasma membrane as a consequence of TrkA activation by NGF [155]. Arf6 is a partner of GGA proteins in regulating the macropinocytosis of APP in lysosomes [156], as well as TrkA and p75NTR trafficking [154,155]. The endocytic cargo-adaptor protein bridging integrator 1 (BIN1) and the scaffolding protein CD2AP are involved in the scission of BACE1 containing recycling carriers from early endosomes [160]. BIN1 also controls presynaptic neurotransmitter release [161], while CD2AP drives TrkA location to endosomes and the TrkA-induced AKT signaling pathway [163]. VPS35 is a critical component of the retromer cargo-recognition complex, which is involved in BACE1 endosome-to-Golgi retrieval transporting to inhibit Aβ production [165]. The Rab family small GTPase proteins, which function as master regulators of vesicular transport and membrane trafficking, have also been implicated in AD pathogenesis (recently reviewed by Zhang X. et al.) [167].

## 6. Targeting the Common Pathway for Preventing Both Amyloidogenesis and Neurotrophic Dysfunction

Research on disease-modifying treatments for AD has largely focused on preventing, eliminating or reducing amyloid plaque accumulation [1,12]. The neurotrophic factor hypothesis for AD pathogenesis supports the idea that targeting NTF signaling pathways is a potential therapeutic tool for AD. The anticipated roles of NTFs (typically NGF and BDNF) in AD treatment have been discussed recently by various labs [28,29,30]. Original research exploring the roles of NTFs on AD was initiated in NGF and has accordingly transitioned to clinical trials [68,69]. Although early clinical trials showed that NGF specifically affected AD patients, NGF cannot pass through the blood–brain barrier (BBB), and direct injection of NGF may cause adverse effects, such as pain and weight loss [2,168,169]. Tuszynski et al. initiated a clinical trial of NGF gene therapy in patients with early-stage AD and confirmed that NGF improves the function of degenerated neurons in the brain tissue of AD patients without obvious side effects [68,69]. Studies using encapsulated cell biodelivery (ECB) of NGF to the cholinergic basal forebrain of AD patients showed an increased NGF concentration at the target area with fewer off-target adverse effects [170,171].

In addition to the strategies directly targeting amyloid plaque accumulation or NFT signaling, the effectors simultaneously regulating both the neurotrophic pathway and amyloidogenic process may be promising targets for AD treatment. Recent research on the intracellular cargo transport deficiencies in AD suggests that improving the efficacy of intracellular trafficking may ameliorate AD pathology [11,119,122]. Recovering the normal expression level via gene therapy or specific drugs could be a choice to treat AD. Researchers have already verified that targeting specific retromers can ameliorate AD pathologies in mouse brain, including amyloid pathology, etc. [172,173,174,175]. Small molecules, such as pharmacological chaperones designed to modulate the stability of retromer complexes, including the VPS35/VPS29 interaction, have been tested for preventing APP cleavage by BACE1 [176,177]. Interestingly, *Vps35* gene delivery into the central nervous system in mice significantly improves synaptic pathology and neuroinflammation, attenuating AD-induced alterations in spatial learning and working memory, significantly reducing Aβ levels and deposition and tau phosphorylation [173]. As the VPS26b/VPS29/VPS35 complex also controls the p75NTR/sortilin interaction, which may affect proNGF signaling, targeting VPS35 levels or the VPS35/VPS29 interaction might be effective in preventing amyloidogenesis and neurotrophic dysfunction simultaneously. On the other hand, the regulators may exhibit activity controlled by phosphorylation, epigenetic modification or GDP/GTP binding, etc. For instance, the phosphorylation status of GGA proteins controls their binding capacity with the ubiquitin tag or sorting signal of target cargo and, thus, decides the sorting fate of cargo. Targeting the activity of the regulators might compensate for the dysfunction results from abnormal expression levels. Therapeutic disease intervention targeting Rab GTPases through prenylation (the modulation of membrane association), GDP/GTP binding or exchange, and the inhibition of protein interactions, etc. has already been suggested [178].

## 7. Conclusions

**Figure 1:**
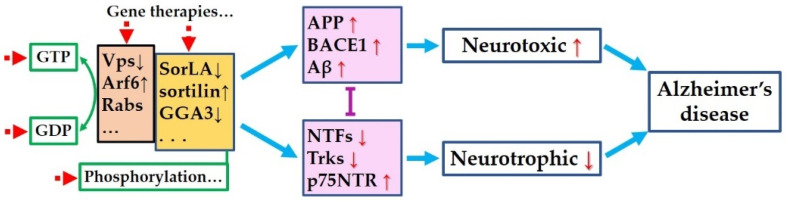
Hypothetical common pathogenesis and targets for Alzheimer’s disease. Amyloidogenic process and neurotrophic pathways are mutually inhibited. Original deficit in any of the two pathways may dysregulate the other one and mutually aggravate each other, or coordinately cause or deteriorate AD. Abnormal expression or activities of the regulators (brown and yellow boxes) result in the dysregulated expression or activities of the key proteins (pink boxes), leads to overloaded neurotoxicity and degraded neurotrophic effects, and finally, causes Alzheimer’s disease. Targeting these regulators (red dotted arrows)—including recover the expression levels or correcting the mutations via gene therapies or specific drugs, or modifying the activity to compensate for the dysfunction resulting from abnormal expression levels or mutation—might be effective strategies to treat AD.

Both the amyloidogenic process and neurotrophic dysfunction account for AD progression. No matter which comes earlier or whether they have a causal link, they possibly share common intracellular regulator deficits in AD. Recovering the expression levels using gene therapy or specific drugs, or modifying the activity of the regulators to compensate for the dysfunction resulting from abnormal expression levels or mutation, might be effective strategies for the treatment of AD (Figure 1).

## Figures and Tables

**Table 1:** Neurotrophic factors and growth factors altered in AD.

Abbreviation	Full Name	In AD
NGF	Nerve growth factor	Mature NGF↓ and proNGF↑ in brain [7,34]; (pro)NGF in hippocampus and neocortex↑ [31]
BDNF	Brain-derived neurotrophic factor	mRNA and protein levels in specific brain areas↓ [29]; decreased BDNF in hippocampus and neocortex↓ [31]; peripheral levels↓ [35]; Val66Met polymorphism [36]
NT-3	Neurotrophin-3	Motor cortex↓ [37]; no change in all brain regions [38]
NT-4/5	Neurotrophin-4/5	Hippocampus and cerebellum↓ [38]
GDNF	Glial cell-line-derived neurotrophic factor	Middle temporal gyrus↓ [39]; serum↓ [40,41]; cerebrospinal fluid↑ and serum↓ [42]; serum and brain of early stage AD↑ [43]
CNTF	Ciliary neurotrophic factor	Increases following brain injury in mice [44]
LIF	Leukemia inhibitory factor	Hippocampus and temporal cortex↑ [45]
CTF-1	Cardiotrophin-1	Brain of APPswe/PS1dE9 mice (AD model)↓ [46]
CDNF	Cerebral dopamine neurotrophic factor	Platelets of probable AD patients↓ [47]
MANF	Mesencephalic astrocyte-derived neurotrophic factor	Inferior temporal gyrus of the cortex↑ [48]
CPE/NF-α1	Carboxypeptidase E/neurotrophic factor-α1	Mutation [33,49]
VEGF	Vascular endothelial growth factor	Prefrontal cortex RNA↑ [50]; frontal cortex and parahippocampal↑ [51]; cerebral capillaries in postmortem brain↓ [52,53]; controversial in cerebrospinal fluid [54,55] and serum [56,57]; 2578C/A and 1154G/A polymorphisms [58]
PDGFs	Platelet-derived growth factors	Controversial in plasma and cerebrospinal fluid [59,60,61]
bFGF	Basic fibroblast growth factors	Brain (Brodmann areas 10/11 and 20/21)↑ [62]
TGFβ1	Transforming growth factors β1	Plasmatic levels↓ [63]; receptor (TGFβRII) in brain↓ [64]
TNF-α	Tumor necrosis factor α	Plasma levels↑; postmortem brain of early-stage AD↑; G308A mutant [65]
CNTF	Ciliary neurotrophic factor	Increased following brain injury in mice [44]
IGF	Insulin-like growth factors	Controversial [66]
HGF	Hepatocyte growth factor	Prefrontal cortex↑ [67]

Note: ↓ indicates upregulated, ↑ indicates downregulated.

**Table 2:** Regulators involved in the amyloidogenic process and the neurotrophic pathways in AD.

Name	Deficits in AD	Effects
ApoE	*ApoE2* and *ApoE4* gene dose-dependent AD risk [123,124,125]	ApoE4 increases Aβ aggregation, synthesis, deposition, reuptake, clearance and degradation [123], while it suppresses BDNF mRNA expression [93].
SorLA	brain↓ [126]; 13 SNPs associated with sporadic AD [127]	APP trafficking in endosomal compartments and Aβ production [121,128,129,130,131,132,133]; Aβ42 degradation [134]; sorting of GDNF and GFRα1/RET [135], trafficking of TrkB [136], activation of the EGFR/ERK/Fos pathway [137].
Sortilin	brain↑ [138]; rs17646665 and other SNPs [139]	BACE1 and APP trafficking [140,141,142]; ApoE/Aβ lysosomal degradation [143,144]; receptor for oligomerized Aβ [145]; anterograde transport of Trk receptors [146]; co-receptor with p75NTR for proNGF [147,148,149], BDNF secretion [150].
GGA3	temporal cortex↓ [151]; gene depletion or rare variant [152]	BACE1 degradation, recycling and axon transport [151,152,153]; TrkA recycling [154]; rapid recruitment of p75NTR to the plasma membrane upon NGF activation of TrkA [155].
Arf6	hippocampus↑ [156]	Regulating macropinocytosis of APP in lysosomes [156]; TrkA post-endocytic recycling [154]; rapid recruitment of p75NTR to the plasma membrane upon NGF activation of TrkA [155].
BIN1	Controversial [157]; SNP rs754834233 [158] and rs138047593 [159]	Endocytic BACE1 recycling [160]; presynaptic neurotransmitter release [161].
CD2AP	rs9349407 [162]	Endocytic BACE1 degradation [160]; TrkA location to endosomes and TrkA-induced AKT pathway [163].
VPS	VPS35 and VPS26 in entorhinal cortex↓ [164]	Promotes BACE1 endosome-to-Golgi retrieval to inhibit BACE1 activation and Aβ production [165]; VPS26b/VPS29/VPS35 retromer complex controls p75NTR/sortilin interaction [166].
Rabs	Rab5, Rab6, Rab7 and Rab10, with abnormal expression or activation	controls amyloidogenesis and the neurotrophic pathway at multiple trafficking stages (recently reviewed by Zhang X. et al.) [167].

## Data Availability

Not applicable.
